# Neural response in obsessive-compulsive washers depends on individual fit of triggers

**DOI:** 10.3389/fnhum.2013.00143

**Published:** 2013-04-22

**Authors:** Ali Baioui, Juliane Pilgramm, Christian J. Merz, Bertram Walter, Dieter Vaitl, Rudolf Stark

**Affiliations:** ^1^Department of Psychotherapy and Systems Neuroscience, Justus Liebig University GiessenGiessen, Germany; ^2^Bender Institute of Neuroimaging, Justus Liebig University GiessenGiessen, Germany; ^3^Outpatient Clinic for Behavior Therapy, Justus Liebig University GiessenGiessen, Germany

**Keywords:** OCD, washers, fMRI, symptom provocation, orbitofronto-striatal network, individualization, contamination, basal ganglia

## Abstract

**Background:** Patients with obsessive-compulsive disorder (OCD) have highly idiosyncratic triggers. To fully understand which role this idiosyncrasy plays in the neurobiological mechanisms behind OCD, it is necessary to elucidate the impact of individualization regarding the applied investigation methods. This functional magnetic resonance imaging (fMRI) study explores the neural correlates of contamination/washing-related OCD with a highly individualized symptom provocation paradigm. Additionally, it is the first study to directly compare individualized and standardized symptom provocation.

**Methods:** Nineteen patients with washing compulsions created individual OCD hierarchies, which later served as instructions to photograph their own individualized stimulus sets. The patients and 19 case-by-case matched healthy controls participated in a symptom provocation fMRI experiment with individualized and standardized stimulus sets created for each patient.

**Results:** OCD patients compared to healthy controls displayed stronger activation in the basal ganglia (nucleus accumbens, nucleus caudatus, pallidum) for individualized symptom provocation. Using standardized symptom provocation, this group comparison led to stronger activation in the nucleus caudatus. The direct comparison of between-group effects for both symptom provocation approaches revealed stronger activation of the orbitofronto-striatal network for individualized symptom provocation.

**Conclusions:** The present study provides insight into the differential impact of individualized and standardized symptom provocation on the orbitofronto-striatal network of OCD washers. Behavioral and neural responses imply a higher symptom-specificity of individualized symptom provocation.

## Introduction

Obsessive-compulsive disorder (OCD) is characterized by recurrent and intrusive thoughts, images, or impulses (obsessions) which often trigger repetitive behaviors (compulsions) such as washing, checking, or mental rituals (American Psychiatric Association, 1994). Despite an ongoing discussion, there is growing clinical (Hasler et al., 2005), factor-analytical (Bloch et al., 2008), neurofunctional (Mataix-Cols et al., 2004), neurostructural (van den Heuvel et al., 2009), and genetic (Hasler et al., 2007) evidence that OCD symptoms can be condensed into distinct subtypes. In a meta-analysis, Bloch et al. (2008) consolidating data from 21 factor analysis studies confirmed a four-factor symptom structure and identified the following subtypes: (1) symmetry/repeating/ordering/counting, (2) forbidden thoughts/checking, (3) contamination/washing, and (4) hoarding. The contamination/washing subtype (contamination obsessions with cleaning/washing compulsions) is one of the most frequent OCD subtypes. Approximately 45–60% of OCD patients suffer from contamination obsessions and/or washing compulsions (Pinto et al., 2006; Matsunaga et al., 2010; Wang et al., 2012). On the one hand, triggers very much vary, are highly idiosyncratic, and often connected with implausible or magical beliefs (Rozin et al., 1986). On the other hand, compulsions are relatively homogenous within this subtype—patients typically feel the urge to reduce the obsessions by means of excessive and ritualistic washing/cleaning compulsions (Markarian et al., 2010). As behavioral studies show, symptom intensity during confrontation with a trigger can be validly operationalized as the “urge to ritualize”; in contamination/washing-related OCD as the “urge to wash hands” (Jones and Menzies, 1997). As opposed to arousal, valence and anxiety ratings, this symptom-specific rating differentiates well between OCD-specific stimuli and generally aversive stimuli in OCD patients (Simon et al., 2010).

The current evidence from functional and structural neuroimaging studies on OCD has been consolidated in an extended *cortico-striatal network model* (Menzies et al., 2008) that integrates brain regions outside the orbitofronto-striatal loop (Saxena et al., 1998). It states that OCD symptomatology is particularly mediated by abnormalities of two relatively segregated fronto-striatal loops: the *affective loop* and the *spatial/attentional loop.* The affective loop includes orbitofrontal cortex, ventral striatum (most prominent structure: nucleus accumbens), ventral pallidum, and mediodorsal thalamus with putative influences from anterior cingulate cortex, hippocampus, and basolateral amygdala. Dysregulation of the affective loop in OCD is assumed to be linked to deficits regarding representation of reward and punishment, anxiety and emotional processing, and in inhibitory control (Menzies et al., 2008). The spatial/attentional loop includes dorsolateral prefrontal cortex (dlPFC), nucleus caudatus, pallidum, thalamus, and substantia nigra and is putatively affected by supramarginal gyri (SMG), angular gyri, ventrolateral prefrontal cortex (vlPFC), and subthalamic nucleus. Dysregulation of the spatial/attentional loop in OCD seems to be related to deficits regarding executive planning, cognitive flexibility, implicit learning, and working memory (Menzies et al., 2008).

Nonetheless, there is a considerable heterogeneity among the results of present functional OCD neuroimaging studies (for reviews see Whiteside et al., 2004; Rotge et al., 2008). The vast majority of these studies so far have investigated samples of OCD patients with different subtypes, neglecting the specificity of the separate subtypes. Structural (van den Heuvel et al., 2009) and functional (Mataix-Cols et al., 2004) neuroimaging studies support the thesis that different brain structures could be involved in the etiology of each subtype. Only few neuroimaging studies have investigated contamination/washing-related OCD by examining this patient group separately (Phillips et al., 2000; Shapira et al., 2003), exclusively (McGuire et al., 1994; Rauch et al., 2002; Chen et al., 2004; van den Heuvel et al., 2004) or by using subtype-specific symptom provocation (Mataix-Cols et al., 2004). These studies pointed out that orbitofrontal cortex (Rauch et al., 2002; Chen et al., 2004), insula (Phillips et al., 2000; Shapira et al., 2003), amygdala (van den Heuvel et al., 2004), thalamus (Chen et al., 2004), pallidum (McGuire et al., 1994), and nucleus caudatus (Chen et al., 2004; Mataix-Cols et al., 2004) are particularly involved in contamination/washing-related OCD.

One way to address the diversity of OCD phenomenology is to use subtype-specific symptom provocation. Mataix-Cols' workgroup published the Maudsley Obsessive-Compulsive Stimuli Set (MOCSS; Mataix-Cols et al., 2009), a *standardized* pictorial stimulus set with subsets for all main OCD subtypes.

However, other researchers tried to account for the idiosyncrasy of OCD by using subject-specific stimuli. This was accomplished by using an *individualized* selection of stimuli from a picture pool according to the patients' ratings of symptom intensity (Simon et al., 2010) or by creating unique individualized stimuli that actually show the personal triggers of each patient (Schienle et al., 2005). In sum, we agree with Simon et al. (2010) that “to account for the phenotypic heterogeneity of OCD, there is a need to use validated *and* individually tailored stimuli.”

In the present fMRI study, we attempted to optimize the investigation of the neural correlates of OCD. Firstly, to reduce complexity of the clinical sample, we investigated contamination/washing subtype only. Secondly, to ensure stimulus specificity and to account for the remaining heterogeneity, we realized a highly individualized symptom provocation paradigm. Thirdly, to allow comparison with previous studies and between-group approaches, we also integrated a standardized and validated symptom provocation approach (MOCSS). We argue that both, subtype-specific standardized as well as subject-specific individualized symptom provocation, have their advantages. Fourthly, in order to test on a theory-driven basis, the regions of interest (ROIs) for this study (see Appendix) are identical to the regions of the current neurobiological model: the ROIs correspond with the two both fronto-striatal loops, without their “putatively influencing regions” (Menzies et al., 2008).

The question to what extent and how both symptom provocation approaches evoke activation in these regions is, however, not only of methodological interest. It is highly relevant for a better understanding and advanced therapy of OCD, because it is able to shed light on the vividly discussed (see Summerfeldt et al., 1999; McKay et al., 2004; Hasler et al., 2005; Mataix-Cols et al., 2005; Matsunaga et al., 2010) interplay of individual and common factors of OCD etiology from a neurobiological perspective.

We hypothesized that our highly individualized symptom provocation approach would evoke heightened activation in regions central to OCD etiology. Therefore, we expected elevated responses in structures of both, the affective loop and the spatial/attentional loop, especially in the basal ganglia, the intersection of both loops. We also hypothesized that when directly comparing both approaches, individualized symptom provocation would induce stronger activation in these structures.

## Methods

### Ethics statement

All procedures are in accordance with the Declaration of Helsinki. This study was approved by the ethical review board of the faculty. The official name of the ethical review board is “Lokale Ethik–Kommission des Fachbereichs 06 der Justus Liebig Universität Gieß en (LEK FB06)” (translation: “Local ethical review committee of the faculty 06 of the Justus Liebig University Giessen”; The faculty 06 is the faculty for psychology and sports science). Procedures and measures were explained to the participants. Written informed consent was obtained from all subjects.

### Participants

Thirty-eight subjects participated in the experiment: 19 subjects suffering from OCD with washing symptoms (“OCD patients”; 12 females; *M*_age_ = 31.78; *SD*_age_ = 7.89; 17 right-handed, one ambidextrous) and 19 healthy controls (“HC”; *M*_age_ = 31.99; *SD*_age_ = 7.46) matched by sex, age and handedness (except for the ambidexter).

None of the patients received psychotherapeutic treatment at the time of the experiment, four patients were completely therapy naïve, five patients were medicated (four SSRIs, one SNRI). Of the remaining 10 patients (which had not received any treatment at the time of the experiment for at least 1 month), 6 had a history of psychotherapeutic and pharmacological treatment, 4 had a history of psychotherapeutic treatment only. The patients' mean illness duration was 12.12 years (*SD* = 9.05 years; range: 1.3–30 years). Five patients had additional Axis I disorders [patient 1: Specific Phobia (heights), patient 2: Major Depression, patient 3: Dysthymia and Generalized Anxiety Disorder, patient 4: Social Phobia, Specific Phobia (spiders), patient 5: Major Depression, partly remitted]. All subjects had a normal or corrected-to-normal vision.

Two clinical psychologists obtained the diagnoses and tested all inclusion and exclusion criteria by using the Structured Clinical Interview for DSM-IV (SCID; Axis I First et al., 1997b; Wittchen et al., 1997; Axis II First et al., 1997a; Fydrich et al., 1997), the Obsessive-Compulsive Inventory-Revised (OCI-R; Foa et al., 2002; Gönner et al., 2007), the Yale-Brown Obsessive-Compulsive Rating Scale and Symptom Checklist (Y-BOCS; Goodman et al., 1989a,b; Hand and Büttner-Westphal, 1991) and the Beck Depression Inventory II (BDI-II; Beck et al., 1996; Hautzinger et al., 2006). Inclusion and exclusion criteria for both groups are described in Table 1.

**Table 1:** **Overview of all inclusion and exclusion criteria for patients and healthy controls**.

**OCD Patients**		**Healthy controls**	
**Inclusion criteria**	**Exclusion criteria**	**Inclusion criteria**	**Exclusion criteria**
OCD as primary diagnosis	Any ICD-10 F0, F1 or F2 diagnosis	Inclusion criteria for the control group were defined as fitting to the respective matching partner by the following criteria: Sex Age (±1 year) Handedness Highest level of education (as far as possible)	The same exclusion criteria as for the patient group
Cutoff for washing subtype in OCI-R reached.	Current psychotherapy		**Plus**
Y-BOCS ≥ 16	Current or prior (1 month) medication with antipsychotics or benzodiazepines		
Illness duration of at least 4 months	Other but unstable (1 month) medication (e.g., with SSRIs)		Any current or past (adulthood) psychological disorder
	Severe depression or suicidal tendencies		Reaching any OCI-R or BDI-II cutoff
	Manic symptoms		Any psychotropic treatment in the past
	PTSD		Any substance abuse in the last 6 months (SCID-I)
	Borderline, antisocial, paranoid, schizoid personality disorder		Job with “ritualized hand washing” (e.g., physician, nurse)
	Neurological illness		
	MRI exclusion criteria		

Additional clinical data were obtained with the trait scale of the State-Trait Anxiety Inventory (STAI-T; Spielberger et al., 1970; Laux et al., 1981) and the Questionnaire for the Assessment of Disgust Sensitivity (QADS; Schienle et al., 2002). The most important clinical data are summarized in Table 2.

**Table 2:** **Overview and test statistics for central clinical data for OCD patients and healthy controls**.

	**OCD patients**		**Healthy controls**		***t*-test^b^**
	**(***n*** = **19**)**		**(***n*** = **19**)**		
	***M***	***SD***	***M***	***SD***	
Y-BOCS score^a^	23.95	4.99	–	–	–
OCI-R	27.53	13.74	3.42	3.49	***
OCI-R washing percentile (OCD population)	51.89	27.95	0.21	0.63	***
OCI-R checking percentile (OCD population)	21.95	29.79	1	2.13	**
BDI-II	15.11	8.70	3.53	2.99	***
STAI-T	51.53	10.69	34.89	7.67	***
QADS	98.58	22.49	76.00	16.44	***

a Meaningful Y-BOCS scores cannot be obtained from healthy controls.

b Three asterisks represent p < 0.001, two asterisks represent p < 0.01.

Patients had moderate (*n* = 12) to severe (*n* = 7) Y-BOCS symptom severity and scored significantly higher than controls on OCI-R, BDI-II, STAI-T, and QADS. Patients scored significantly higher on the washing subscale than on the checking subscale of the OCI-R (mean percentiles/OCD population; paired *t*-test; *T*_(18)_ = 4.237; *p* < 0.001). The german OCI-R offers no mean percentiles (OCD population) for the other subtypes (Gönner et al., 2009, p. 49, footnote 9). As inclusion criteria defined, all patients reached the OCI-R cutoff for washing and no member of the HC group reached any cutoff. OCI-R cutoffs of other subtypes were reached by several patients (checking: 11 obsessions: 6, mental neutralizing: 11, ordering: 2, hoarding: 3). Patients scored significantly higher on the washing subscale than on the checking subscale of the OCI-R (mean percentiles/OCD population; paired *t*-test; *T*_(18)_ = 4.237; *p* < 0.001). All subjects received €8 per hour for participation.

### Stimuli and design

**Figure 1:**
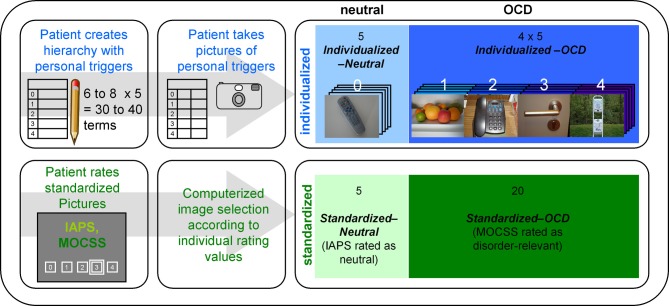
**Overview of the stimulus creation procedure carried out with every patient.** The upper row represents the creation process for the individualized pictures, while the lower row represents the selection process for the standardized pictures. IAPS stands for “International Affective Picture System” (Lang et al., 2008); MOCSS stands for “Maudsley Obsessive-Compulsive Stimuli Set” (Mataix-Cols et al., 2009). Darker color shades represent higher stimulus intensities. The photographs depicted here are pictures taken by a participant of the OCD group (written informed consent for publication has been obtained). The white numbers above the photographs represent the stimulus intensity (the hierarchy level) of each picture. “Computerized image selection” refers to the image selection process described in the *Stimuli and Design* section.

For every patient (but not for healthy controls), an *Individualized* stimulus set was created in a multi-step method (see Figure 1). First, patients were given a blank table with five rows representing five levels of trigger intensity and were instructed to create a personal OCD hierarchy with 6–8 triggers per row concentrating on triggers of their daily lives. Trigger intensity was operationalized as “urge to wash hands” ranging from “0—no urge” to “4—very strong urge.” Participants were instructed to imagine touching the object with their hands. Assistance was given only if necessary and by means of verbal stimulation (e.g., “imagine a normal day,” “think of what you touched yesterday”). Patients were not informed about the reason (see next paragraph) for the creation of a hierarchy until its completion. By this, it was intended to prevent that patients avoid naming highly aversive triggers. The reason for this was explained to all subjects at the end of the stimulus creation procedure.

After completion of the hierarchy, they were instructed to photograph these triggers according to a detailed protocol. The resulting photographs were screened for insufficient quality and other exclusion criteria (e.g., showing faces or other details revealing the identity of the patient). Surplus pictures were randomly deleted. Five pictures from the lowest level of the hierarchy (“0—no urge”) formed the *Individualized-Neutral* condition, while 20 pictures from other levels formed the *Individualized*-*OCD* condition.

For the *Standardized* stimulus set, patients had to rate 30 pictures from the “washing subset” of the Maudsley Obsessive-Compulsive Stimuli Set (MOCSS, Mataix-Cols et al., 2009) and 40 neutral pictures (see Appendix) taken from the International Affective Picture System (IAPS; Lang et al., 2008) with the same instructions and scales as for the individual hierarchy. This procedure was conducted in order to prevent the inclusion of non-neutral IAPS pictures. It also prevented non-disorder-relevant MOCSS pictures entering the final stimulus set. Thus, the second stimulus set consisted of 20 MOCSS pictures rated as disorder-relevant (*Standardized-OCD*) and five neutrally rated IAPS pictures (*Standardized-Neutral*).

### Procedure

For the patient group, the experiment consisted of 3 sessions on 3 different days. The first session comprised OCD specific diagnostics and questionnaires, creation of the OCD hierarchy, rating of the standardized pictures, instructions for photographing the personal OCD triggers, and an anatomical MRI scan. Between the first and the second session, patients took photographs of their personal OCD triggers. At the second session, patients handed over the camera with the photos and the respective filled out protocols. Then, a complete SCID (Axes I and II) was conducted.

The third session consisted of the actual symptom provocation experiment. Again, the instruction included the prompt to imagine oneself touching the object and to rate the urge to wash hands on a scale ranging from “0—no urge” to “4—very strong urge.” The experiment consisted of two uninterrupted runs with 50 trials each. All pictures were presented in a pseudo-randomized order with no more than two pictures of each stimulus set (Individualized, Standardized) in a row.

Each trial started with a black screen shown for 0–2.375 s, followed by the presentation of a picture for 5 s. This was followed by the presentation of a black screen for 1.5 s and the rating scale for 5 s. The inter-trial interval (ITI) was 6.625–8.5 s. A fixation cross was presented in the center of the screen during the ITI. Stimuli were projected onto a screen at the end of the scanner (visual field = 18°) and were viewed through a mirror mounted on the head coil. Altogether, the symptom provocation experiment took 33.5 min. After that, participants filled out a questionnaire concerning emotional experiences during the experiment.

The procedure was adapted for the healthy controls. Diagnostics contained no Y-BOCS and there was no stimulus creation procedure, because healthy controls were confronted with exactly the same pictures as their respective matching partners. Thus, the entire experimental procedure was carried out in only two sessions with the anatomical MRI scan being performed directly before the symptom provocation experiment. Except these points, the procedure was identical.

### Image acquisition

Functional and anatomical scans were obtained using a 1.5 T whole body tomograph (Siemens Symphony) with a standard head coil. Structural image acquisition consisted of 160 T1-weighted sagittal images (MPRage, 1 mm slice thickness). A gradient echo field map sequence was acquired before the functional image acquisition to obtain information for unwarping B_0_ distortions. For functional images, a total of 810 whole-brain images were registered using a T2^*^-weighted gradient echo-planar imaging sequence (EPI) with 25 slices [slice thickness = 6 mm, including 1 mm gap; descending slice order; *TR* = 2.5 s, *TE* = 55 ms, *TA* = 100 ms, flip angle = 90°, field of view = 192 × 192 mm; matrix size = 64 × 64; voxel size = 3 × 3 × 6 mm (including the gap)]. The orientation of the axial slices was tilted to parallel the orbitofrontal cortex tissue–bone transition in order to reduce susceptibility artifacts in the orbitofrontal cortex (see Deichmann et al., 2003).

### Data analysis

Behavioral data were analyzed with SPSS for Windows (Release 19.0; IBM) using analyses of variance (ANOVA) of rating scores averaged by person. ANOVA were computed with three independent factors: *Group* (OCD patients, HC), *Disorder Relevance* (OCD relevant/neutral) and *Stimulus Set* (Individualized/Standardized). Pearson correlations were computed with unaveraged data in order to check overall consistency of ratings across experimental phases.

Imaging data were analyzed using Statistical Parametric Mapping (SPM8, Wellcome Department of Cognitive Neurology, London, UK; 2008) implemented in Matlab R2007b (Mathworks Inc., Sherborn, MA). For a detailed description of fMRI data processing and analysis, see Appendix. Briefly, preprocessing comprised outlier detection (see Appendix), B_0_ unwarping and realignment to the first volume (b-spline interpolation), slice time correction, co-registration, and normalization to the standard space of the Montreal Neurological Institute brain (MNI-brain). Resolution after normalization was 3 × 3 × 3 mm. Finally, EPI images were spatially smoothed (Gaussian kernel; FWHM = 9 mm).

*Individualized-OCD* pictures were tested against *Individualized-Neutral* pictures (IND). Standardized symptom provocation (STD) was computed by testing *Standardized-OCD* against *Standardized-Neutral*. Direct comparisons between both symptom provocation approaches (IND vs. STD) were computed by contrasting IND (*Individualized-OCD* minus *Individualized-Neutral)* with STD (*Standardized-OCD* minus *Standardized-Neutral)* on the first-level, using a double contrast vector. All contrasts were computed on the first-level for all subjects and then used in the second-level analyses. Within-group contrasts were tested using one-sample *t*-tests, between-group contrasts (OCD patients vs. HC) were analyzed with two-sample *t*-tests.

For all models and contrasts, ROI analyses were carried out using the small volume correction option of SPM8. Used ROI masks (for further information see Appendix) comprised only the regions of the cortico-striatal network model, without the “putatively influencing regions” (Menzies et al., 2008). The significance threshold was set to α = 0.05 on voxel level tests, corrected for multiple testing family-wise-error correction (FWE). Minimum cluster size was set to 5 voxels.

In order to explore the nature of a possible relationship between the intensity of the triggers (as defined by the hierarchy level) and the neural responses, an additional exploratory analysis of the individualized pictures was conducted. Since there is no rationale to decide whether to test for linear or non-linear relationships, we computed an *F*-test, in order to check for significant variance differences across hierarchy levels in all ROIs. The methods used for this purpose were the same as described above and in the Appendix, except that a factorial model was built in order to conduct the *F*-test.

## Results

### Ratings

**Figure 2:**
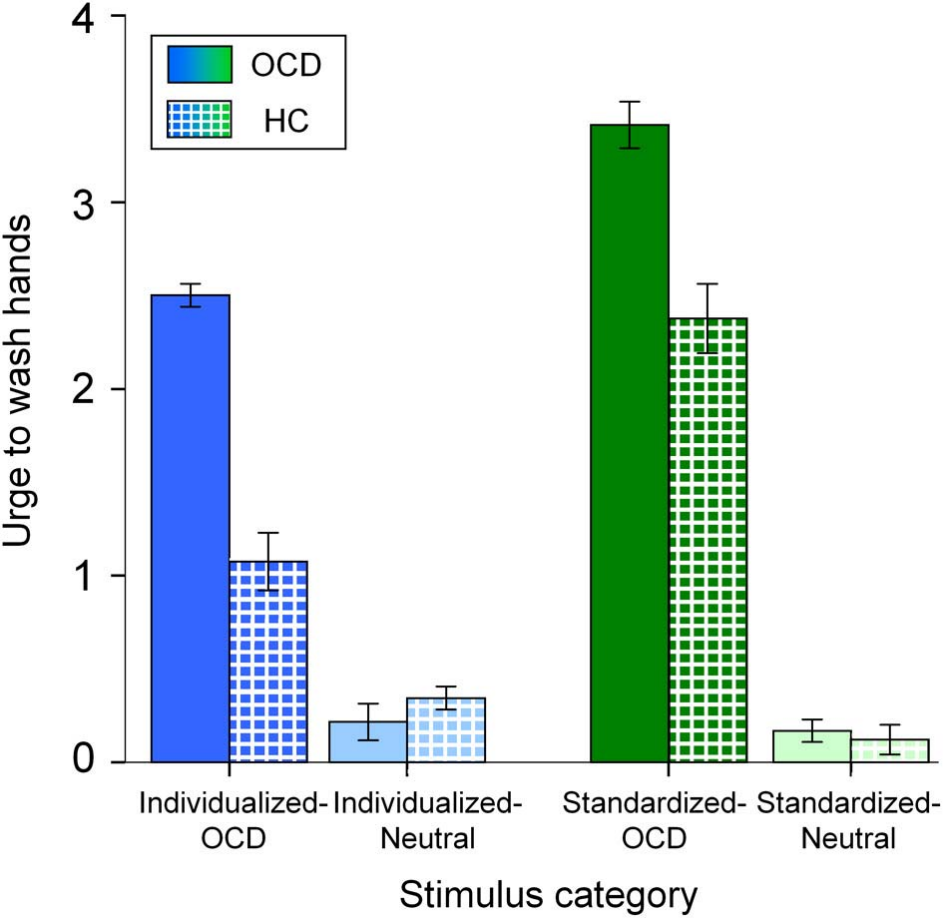
**Mean urge to wash hands (and standard errors of the mean) of patients (OCD; solid colors) and healthy controls (HC; patterned) for all stimulus categories.** Note that the different colors depict the stimulus categories (dark blue: *Individualized-OCD*; light blue: *Individualized-Neutral*; dark green: *Standardized-OCD*; light green: *Standardized-Neutral;* also cp. Figure 1).

Mean (+*SE*) ratings of “urge to wash hands” are depicted in Figure 2. A main effect for Disorder Relevance shows that OCD pictures were generally rated as provoking stronger urges to wash hands than neutral pictures [*F*_(1, 144)_ = 718.555, *p* < 0.001]. As a main effect for Group reveals, overall, patients had higher ratings than controls [*F*_(1, 144)_ = 56.250, *p* < 0.001]. A main effect for Stimulus Set shows higher ratings for standardized than for individualized pictures [*F*_(1, 144)_ = 37.416, *p* < 0.001]. Ratings showed significant interactions for Disorder Relevance by Stimulus Set [*F*_(1, 144)_ = 61.056, *p* < 0.001] and Group by Disorder Relevance [*F*_(1, 144)_ = 64.069, *p* < 0.001]. The interaction Stimulus Set by Group was not significant [*F*_(1, 144)_ = 4.075, *p* = 0.5]. The three way interaction Group by Disorder Relevance by Stimulus Set was marginally significant [*F*_(1, 144)_ = 3.154, *p* = 0.078]. As can be seen in Figure 2, this three way interaction can be explained by individualized pictures being more effective in differentiating between OCD patients and HC than standardized pictures.

### FMRI data

**Figure 3:**
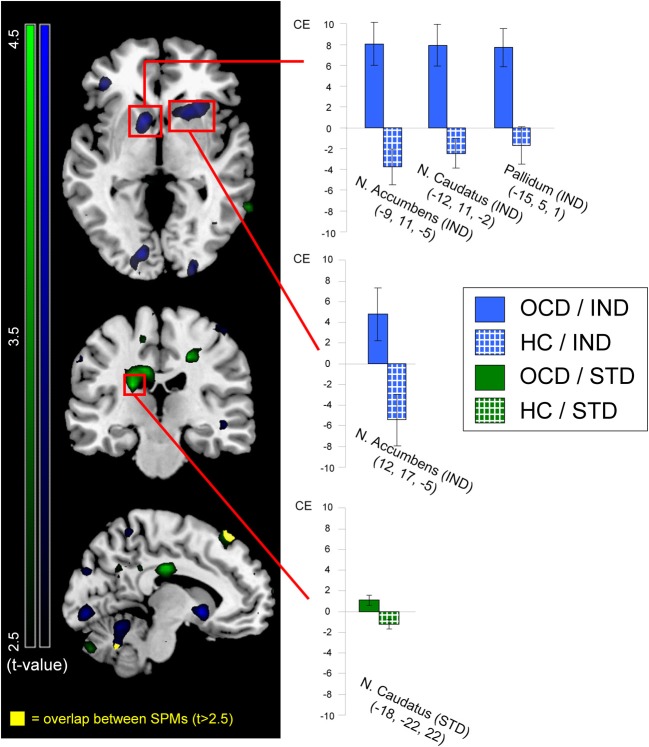
**Neural activation of patients (OCD; solid colors) greater than healthy controls (HC; patterned) contrasted for individualized (IND; blue) and standardized (STD; green) symptom provocation.** The figure displays statistical parametrical maps with whole-brain *t*-values for the between-group contrasts (OCD > HC) for both symptom provocation approaches. For illustration reasons, data were thresholded with *t* > 2.5 (see color bars for exact *t*-values) and displayed on a standard MNI brain template. Significant results from the voxel-wise ROI analyses are marked with red rectangles. Additionally, all significant between-group results are further depicted using the peak voxels of the OCD group: the bar graphs illustrate mean contrast estimates (CE) of the symptom provocation contrasts (with the corresponding standard errors of the mean) for patients (gray) and healthy controls (white). All coordinates are given in MNI space. The lower slice (*x* = −10; left hemisphere) depicts the only regions with an overlap (yellow) between both whole-brain statistical parametrical maps, with a threshold of *t* > 2.5; these regions were not included in any ROI and are depicted for illustrative purposes only.

Figure 3 displays neural activation between OCD and HC, separately for individualized (blue) and standardized symptom provocation (green).

Significant ROI activations for both symptom provocation approaches are summarized in Table 3.

**Table 3:** **Within-group and between-group results of ROI analyses for both standardized and individualized symptom provocation**.

**Contrast**	**Region**	**OCD**							**HC**						**OCD > HC**					
		**Side**	**Size**	***x***	***y***	***z***	*****T***_**max**_**	*****p***_**corr**_**	**Size**	***x***	***y***	***z***	*****T***_**max**_**	*****p***_**corr**_**	**Size**	***x***	***y***	***z***	*****T***_**max**_**	*****p***_**corr**_**
IND	N. Accumbens	L	26	−9	11	−5	3.87	0.012							30	−9	11	−5	4.38	0.001
	N. Accumbens	R													20	12	17	−5	2.83	0.039
	N. Caudatus	L	36	−9	11	−2	3.99	0.036							35	−12	11	−2	4.28	0.007
	Pallidum	L	24	−15	5	−2	4.41	0.010							21	−15	5	1	3.62	0.018
STD	Angular G.	L	91	−57	−61	16	4.22	0.037	31	−39	−52	46	4.71	0.016						
	N. Caudatus	L													6	−18	−22	22	3.52	0.035
	Thalamus	L	90	−12	−31	−5	4.45	0.028												

All coordinates are given in MNI space. The threshold was p_corr_ = 0.05 (FWE-corrected according to SPM8; for ROIs: small volume correction; L = left; R = right). No ROI was significantly more strongly activated in the HC compared to the OCD group.

### Individualized symptom provocation

The contrast IND showed ROI activation of nucleus accumbens, nucleus caudatus and pallidum in the OCD group. In the HC group, no suprathreshold ROI activation was found. Between-group tests demonstrated greater activation in OCD patients compared to HC in nucleus accumbens, nucleus caudatus, and pallidum. No ROI was significantly more strongly activated in the HC compared to the OCD group.

### Standardized symptom provocation

ROI analyses for the contrast STD showed activation in angular gyrus and thalamus in the OCD group. In the HC group, ROI activation was found in angular gyrus. Between-group tests showed greater activation in the OCD compared to the HC group in the nucleus caudatus. No region was significantly more strongly activated in the HC than in the OCD group.

### Individualized vs. standardized symptom provocation

Significant ROI activations for the comparison of both symptom provocation approaches are summarized in Table 4.

**Table 4:** **Within-group and between-group results of ROI analyses for the computational comparisons of both symptom provocation approaches**.

**Contrast**	**Region**	**OCD**							**HC**						**OCD > HC**					
		**Side**	**Size**	***x***	***y***	***z***	*****T***_**max**_**	*****p***_**corr**_**	**Size**	***x***	***y***	***z***	*****T***_**max**_**	*****p***_**corr**_**	**Size**	***x***	***y***	***z***	*****T***_**max**_**	*****p***_**corr**_**
IND > STD	N. accumbens	L	10	−12	17	−8	3.42	0.025							27	−12	17	−5	3.57	0.009
	N. accumbens	R													8	12	20	−5	3.12	0.020
	N. caudatus	L													54	−12	14	−2	3.47	0.040
	N. caudatus	R													43	15	20	−2	3.77	0.020
	Pallidum	L													17	−15	5	−5	3.47	0.023
STD > IND	Angular g.	R	47	48	−58	19	4.27	0.046												
	N. accumbens	L							22	−12	8	−8	3.72	0.015						
	N. accumbens	R							7	12	20	−5	3.15	0.032						

All coordinates are given in MNI space. The threshold was p_corr_ = 0.05 (FWE-corrected according to SPM8; for ROIs: small volume correction; L = left; R = right).

The comparison IND > STD showed ROI activation in nucleus accumbens in the OCD group but no significant ROI activation in HC. The between-group analysis showed greater activation in nucleus accumbens, nucleus caudatus, and pallidum for the OCD group compared with HC.

The opposite contrast (STD > IND) was accompanied by stronger BOLD responses in angular gyrus in the OCD group. In the HC group, significantly stronger activation was found in the nucleus accumbens.

### Exploratory analysis of OCD hierarchy levels

**Figure 4:**
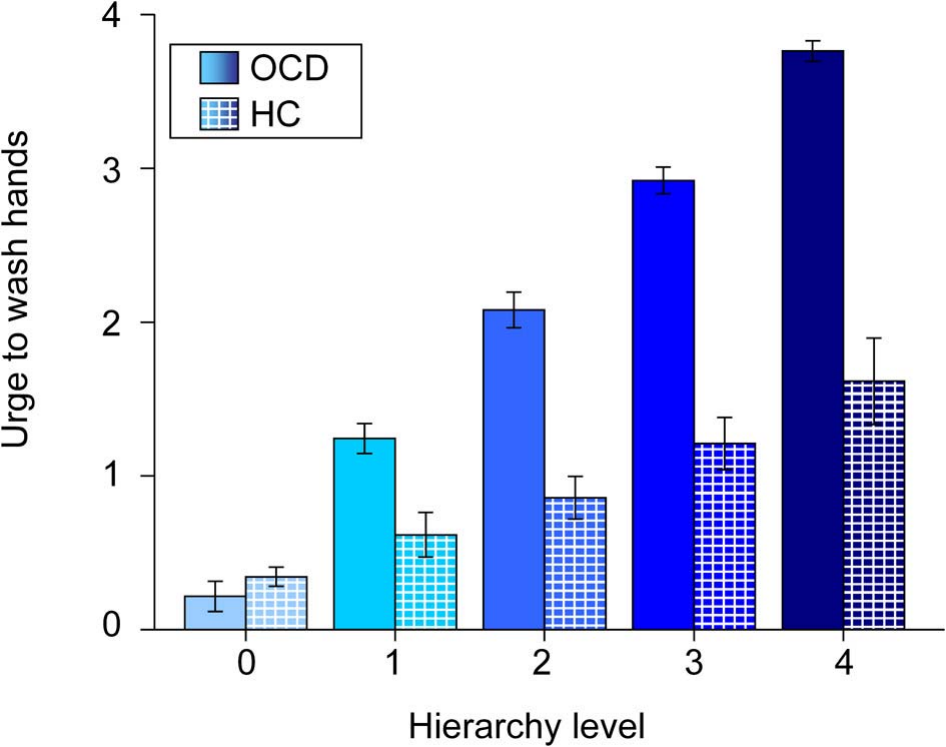
**Mean urge to wash hands (and standard errors of the mean) of patients (solid colors) and healthy controls (patterned) for individualized pictures plotted against hierarchy levels.** Note that values of healthy controls are plotted against the original hierarchy level values of their respective matching partners. The different shades of blue represent the different original hierarchy level values (cp. Figure 1).

Patients rated symptom intensities with a high consistency across the experimental phases. Ratings of *Individualized* pictures during the fMRI experiment and their prior grading in the hierarchy were highly correlated (*r* = 0.87; *p* < 0.001). There was also a positive correlation between the subjective ratings HC have given to the individualized pictures of their respective matching partners and the original hierarchy levels (from the OCD group) of these stimuli (*r* = 0.37; *p* < 0.001). The relationship between hierarchy level and subjective ratings is depicted in Figure 4.

**Figure 5:**
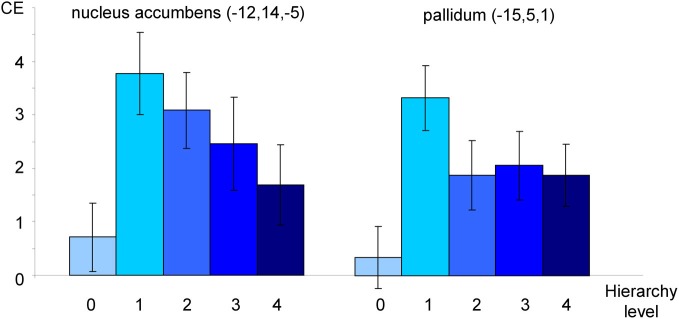
**Neural activation of patients toward individualized pictures plotted against hierarchy levels.** Mean contrast estimates (CE; and standard errors of the mean) in peak voxels in nucleus accumbens and pallidum as identified in an *F*-Test (see text). All coordinates are given in MNI space. The different shades of blue represent the different original hierarchy level values (cp. Figure 1).

The exploratory ROI analysis showed variance differences across hierarchy levels in neural activation in the OCD group in nucleus accumbens (*x* = −12, *y* = 14, *z* = −5; *T*_max_ = 5.14; *p*_corr_ = 0.021) and pallidum (*x* = −15, *y* = 5, *z* = 1; *T*_max_ = 5.61; *p*_corr_ = 0.025). The relationships between the hierarchy levels and neural activation in the two structures are depicted in Figure 5.

## Discussion

The main goal of the present study was to examine the neural correlates of obsessive-compulsive washers by means of a highly *subject*-specific individualized symptom provocation paradigm. Additionally, it aimed at comparing this procedure with an existing *subtype*-specific symptom provocation paradigm (MOCSS).

As the key finding, individualized symptom provocation evoked activity in the main regions of the orbito-frontal network. It also differentiated well between patients and HC, based on neuronal and behavioral data. Standardized symptom provocation showed only little overlap with individualized symptom provocation and did not show comparable differentiation capabilities. The direct comparison of the two symptom provocation approaches underlines the divergence of activation provoked by the two sets of stimuli. Regarding the discussion of neural responses, we will concentrate on between-group results in order to focus on the neural correlates specific for OCD.

Considering the behavioral data, disorder relevant pictures of both stimulus sets provoked higher urges to wash hands than neutral pictures. Overall, *Standardized-OCD* pictures evoked stronger urges than *Individualized-OCD* pictures. This is line with our expectations, since *Individualized-OCD* had been constructed in terms of covering all symptom intensity levels, resulting in a rated mean intensity very close to the scale's mean (of disorder relevant pictures) of 2.5 in the OCD group (see Figure 2; *M* = 2.501; *SD* = 0.26). The marginally significant *Group* by *Disorder Relevance* by *Stimulus Set* interaction points to a potentially stronger differentiation of patients and HC by the *Individualized* stimulus set. This provides evidence for a higher disorder specificity of individualized pictures.

### Individualized symptom provocation

During individualized symptom provocation, nucleus accumbens, nucleus caudatus, and pallidum were significantly more strongly activated in the OCD group compared to HC. These results are largely in accordance with the current neurobiological model, assuming a dysfunction of the orbitofronto-striatal network in OCD patients (see Deckersbach et al., 2002; Menzies et al., 2008). They are also in line with previous studies that examined contamination/washing-related OCD regarding pallidum (McGuire et al., 1994) and nucleus caudatus (Chen et al., 2004; Mataix-Cols et al., 2004).

Schienle et al. (2005) reported greater activation in insula, nucleus caudatus, SMG, thalamus, and prefrontal cortex when contrasting symptom provocation in OCD patients and HC. The present study was able to partly replicate this previously reported pattern; yet we used partly different ROIs. We assume that the additional brain regions that were significantly more strongly activated in our study (pallidum and nucleus accumbens) possibly are due to a stronger statistical power (due to a larger sample) or are more specific to the contamination/washing-related subtype. Indeed, the pallidum has previously been reported in the context of this subtype (McGuire et al., 1994). Heightened activation of the nucleus accumbens during symptom provocation can be understood in the context of the orbitofronto-striatal network as a mediator between orbitofrontal cortex and pallidum within the affective loop (see Menzies et al., 2008). Alternatively, as Sturm et al. (2003) speculate, OCD might even be explained as a dysfunction of the nucleus accumbens due to its “gating” function for both the fronto-striatal and the hippocampo-striatal circuitry. Clinical significance of heightened nucleus accumbens activity has already been shown in several studies using deep brain stimulation as treatment for refractory OCD (for recent reviews see Greenberg et al., 2010; Schlaepfer and Bewernick, 2011).

### Standardized symptom provocation

OCD patients showed heightened activation of nucleus caudatus during standardized symptom provocation, compared with HC, partially replicating previous results (Mataix-Cols et al., 2004). The key role of the nucleus caudatus in OCD etiology has been described in numerous OCD neuroimaging studies (for reviews see Whiteside et al., 2004; Chamberlain et al., 2005; Deckersbach et al., 2006; Friedlander and Desrocher, 2006; Huey et al., 2008). This structure is at the core of the spatial/attentional loop of the orbitofronto-striatal network model (Menzies et al., 2008). Dysfunction of the nucleus caudatus is assumed to be associated with an overfunctioning error detection system in OCD (Guehl et al., 2008), which could be the cause for the “not just right experiences” that are commonly reported (see Coles et al., 2003).

### Comparison of individualized and standardized symptom provocation

A comparison of both symptom provocation approaches raises several questions that need to be discussed.

Behavioral data showed that healthy controls experienced standardized pictures as evoking much stronger urges than their matching partners' individualized pictures. This result is not surprising; the first publication using the MOCSS washing subset was also able to show significant behavioral and neural reactions in healthy controls (Mataix-Cols et al., 2003). The authors stated that their standardized symptom provocation approach can reliably induce OCD-like symptoms in normal subjects (Mataix-Cols et al., 2003). In other words, the MOCSS was constructed to be universally provocative, allowing the investigation of OCD-like symptoms in healthy subjects. We argue that the processes addressed by this approach are nevertheless important in OCD pathology because they are more pronounced in OCD patients (Mataix-Cols et al., 2004); yet they are not exclusive for OCD. The impact of these stimuli on healthy controls might be explained by their content addressing a non-idiosyncratic (“common”) factor of contamination obsessions that might be connected to the processing of basic emotions. Mataix-Cols et al. (2003, 2004) explain the results evoked by the MOCSS washing subset with disgust processing. Unfortunately, the authors have not published the disgust rating data for the MOCSS (Mataix-Cols et al., 2003, 2004). Yet in healthy subjects, the neural activation evoked by the washing subset and a “normally aversive/disgusting” control condition did not differ significantly, except in the middle temporal gyrus and the orbitofrontal cortex (Mataix-Cols et al., 2003).

Behavioral data also showed that individualized symptom provocation did not provoke urges as strong as standardized symptom provocation in OCD patients. This is due to the stimulus creation procedure for the individualized symptom provocation, which was intended to represent the whole spectrum of symptom intensity. On the one hand, this is a major strength of the present study and has allowed the exploration of possible relationships between stimulus intensity and neural responses. On the other hand, the differences in stimulus intensities between both picture sets can be seen as a confounding variable.

Taken together, this leads us to the conclusion that healthy subjects might have reacted strongly toward standardized stimuli because these are more prone to evoke disgust processing. While an influence of the differences in stimulus intensities cannot be excluded, we conclude that there are also qualitative differences between the two symptom provocation approaches. The individualized symptom provocation approach used in the present study is an advancement of an approach developed at our institute by Schienle et al. (2005). There, the individualized stimuli were rated with respect to the induced anxiety and disgust. Healthy controls did not rate the individualized pictures as disgust-inducing or fear-evoking; both mean scores were at the lowest end (“not at all”) of the scale (Schienle et al., 2005). Together with the evidence from MOCSS studies (see above), this suggests a qualitative difference between the two approaches regarding their potential to evoke basic emotions or symptom-like states in healthy subjects. This view is also supported by the direct computational comparison of the two approaches (between-group results) in the present study. Individualized symptom provocation led to significantly stronger reactions in regions that are central in OCD etiology (nucleus accumbens, nucleus caudatus, and pallidum) than standardized symptom provocation. In OCD, dysfunction of the nucleus accumbens has been associated with dysfunctional motor control and emotion processing (Schlaepfer and Bewernick, 2011). Nucleus caudatus as a central region of the spatial/attentional loop, has been assumed to be closely linked to OCD-related deficits regarding executive planning (Menzies et al., 2008). Speculatively, a qualitative difference between the two approaches could be due to individualized pictures being more provocative regarding motor-related aspects. It is plausible to assume that the patients' unique personal triggers might have a stronger implicitly learned association with motor-related reactions than standardized stimuli. Additionally, in the present study subjects were prompted to rate their “urge to wash hands”; this might have intensified a mental preparation of subsequent compulsive behavior.

Moreover, possible differences in the properties of the different individualized picture sets might be seen as a confounding variable. We tried to account for some of this variance in the (e.g., visual) properties by using matched healthy controls. It can be argued that confronting healthy controls with these different individualized picture sets raises the concern of additional inter-subject variance (e.g., through differences in the arousal evoked by these picture sets). Yet, such variance seems inevitable whenever using individualization: symptom specificity is bought at the expense of variance in stimulus properties.

Interestingly, healthy subjects did not show any significant neural responses toward the individualized stimuli of their respective matching partners. On the one hand, this underlines the symptom specificity of the individualized approach. Behavioral data (see Figure 4) shows that healthy respond slightly (but significantly) stronger to the intense individualized pictures of their respective matching partners than to neutral pictures. On the other hand, this lack of significant neural response toward individualized stimuli in the healthy controls might have led to an overestimation of the reported between-group activation. This might be a result of a lack of salience and familiarity of these stimuli for healthy controls. This shortcoming partly lies in the nature of individualization (Schienle et al., 2005).

### Interplay with previous neuroimaging studies on contamination/washing-related OCD

To our knowledge, there are only six neuroimaging studies that have so far investigated groups of OCD washers separately or exclusively (McGuire et al., 1994; Phillips et al., 2000; Rauch et al., 2002; Shapira et al., 2003; Chen et al., 2004; van den Heuvel et al., 2004).

The present results (from both approaches) replicate findings regarding angular gyri (Phillips et al., 2000), n. caudatus (McGuire et al., 1994; Chen et al., 2004), thalamus (McGuire et al., 1994; Chen et al., 2004) and pallidum (McGuire et al., 1994); yet, not regarding OFC (Rauch et al., 2002; Chen et al., 2004).

With the cortico-striatal network model (Menzies et al., 2008) in mind, especially the absence of significant results for the OFC seems to raise questions. However, considering the abovementioned previous studies, only two out of six reported heightened OFC activity—a small to medium sized effect is not likely to be replicated in a study with a medium sample size.

### Exploratory analysis of OCD hierarchy levels

The analysis of the hierarchy levels is interesting for several reasons.

Firstly, behavioral data show that during symptom provocation individualized stimuli were rated as being as intense as during the creation of the hierarchy. The applied individualized symptom provocation approach can be seen as effective in provoking symptoms in the intended “dosage.”

Secondly, the analysis revealed significant variance differences across hierarchy levels in nucleus accumbens and pallidum. In other words, these structures seem to be sensitive towards stimulus intensity. The reported results, together with a visual inspection of contrast estimates (see Figure 5) point at possible non-linear relationships. Non-linear responses to stressful stimuli have been reported in different areas of research investigating stress-related processing and learning (for a review see Baldi and Bucherelli, 2005). A possible explanation for such a response pattern could be that the respective structures have a “decisive function” in OCD processing; in detail, they might regulate how stimuli are processed depending on their level of intensity. Recently, the pallidum has been described as the structure responsible for shifting between the two fronto-striatal loops in OCD (van den Heuvel et al., 2010). Speculatively, the pallidum serves as an intensity detector that decides which regions need to be recruited in order to cope with a stimulus. We must, however, emphasize that these interpretations are only speculative.

## Limitations

The present study has several limitations. Firstly, some psychotropic drugs and some comorbid psychological disorders were allowed in the OCD group (see Table 1). Secondly, the two stimulus sets were not equal regarding stimulus intensity. This was mainly due to the stimulus creation procedure which, on the other hand, also represents a major strength of this study. The individualized picture set was intended to represent the whole spectrum of symptom intensity and to allow for an exploratory analysis of *OCD hierarchy levels*.

One might ask why control subjects did themselves not create individualized stimuli but saw pictures of their corresponding matching partners. It is important to state that in this study, the function of the control group lies mainly in controlling for the neural activation induced by the visual stimulation. We suppose that creating stimulus sets that include “triggers” that evoke very strong urges in healthy controls would have led to extremely disgust-inducing pictures. The use of such material would have led to a comparison between neural correlates of OCD-related processing and general disgust processing; this would have been a poor operationalization of our research question.

The acquisition parameters of the present study have to be acknowledged as an additional limitation. The low resolution of the EPI images is due to a combination of factors: a whole-brain field of view and a (design-typical) low TR on an MRI scanner with relatively low magnet strength (1.5 T). Another methodological limitation is the number of ROIs used for our analyses. Yet, the choice of ROIs was based on a current model (Menzies et al., 2008) and their origin is published in the Appendix.

## Conclusions

From a neurobiological perspective, the present study emphasizes the importance of considering the idiosyncrasy of OCD. Behavioral and neural responses point to a higher symptom-specificity of individualized symptom provocation.

In addition, the presented results contribute to a better understanding of the interplay of individual and common factors of OCD. Firstly, the results show that the degree of individuality of OCD triggers makes a difference to the “OCD brain.” Secondly, they show that only a confrontation with highly *individual* triggers evoke activation patterns that are considered as the *common* neural basis of OCD. We argue that the question about the difference in neural mechanisms behind different OCD subtypes can only be answered if symptom provocation is performed with a high degree of symptom specificity. Speculatively, the diverse neural activation reported in earlier studies for different OCD phenomenology (cp. Mataix-Cols et al., 2004) might just reflect the variance produced by an unspecific symptom provocation approach. In other words: the content validity of symptom provocation might be determined by the fit of the stimuli to the highly diverse individual triggers—the better the content validity, the smaller the “signal noise,” thus the better the ability to depict *a common neural pathway*.

Besides this methodological perspective, which is concerned with the symptom-specificity of the approaches, there are also some etiological and clinical conclusions that can be drawn from the presented results.

There is evidence that standardized symptom provocation, with its more universally provocative triggers, might address processes of OCD etiology that are not unique to OCD, namely the processing of basic emotions. We argue that both approaches trigger different aspects of OCD-related stimuli processing; yet, standardized symptom provocation might more likely provoke processing that OCD shares with other disorders such as specific phobias or with non-pathological processing of anxiety and disgust. This interpretation is also supported by the similarity in neural activation patterns found in the healthy control group and in the OCD group during standardized symptom provocation. Individualized symptom provocation, on the other hand, seems to be more eligible to provoke activation in the spatial/attentional loop. This might be associated with the individualized triggers having a stronger impact on motor-related aspects of OCD. The very unique individual triggers of each patient most likely have a rather strong learned association with subsequent compulsive behavior. Possible clinical relevance becomes obvious if symptom provocation is seen as a model for *Exposure and Response Prevention* (ERP). The success of ERP depends on a sufficient activation of the neural circuitry that is supposed to habituate—in OCD mainly the basal ganglia (Nakatani et al., 2003). We argue that depending on the individual fit of triggers different neural circuits will habituate. That is, triggers with a high degree of individualization might be more eligible to make possible the habituation of compulsive behavior (response prevention), due to a higher association with motor-related aspects of OCD, while universally provocative triggers might be more eligible to make possible the habituation of heightened anxiety and disgust sensitivity. Again, these interpretations are rather speculative.

Ultimately, this is not meant to argue against standardized symptom provocation; this study is merely a first attempt to address the diversity of OCD phenomenology in two different ways and to compare the two approaches within one experimental paradigm. We argue that both, subtype-specific and subject-specific symptom provocation, contribute significantly to our understanding of psychological disorders. In the end, the understanding (and the treatment) of a disorder must always be based on common and individual factors.

### Conflict of interest statement

The authors declare that the research was conducted in the absence of any commercial or financial relationships that could be construed as a potential conflict of interest.

## Appendix

### Materials

The following IAPS pictures were used as a pool for the *Standardized-Neutral* set: 5001, 5020, 5030, 5201, 5210, 5220, 5470, 5551, 5593, 5594, 5600, 5611, 5631, 5661, 5665, 5725, 5726, 5740, 5750, 5760, 5779, 5780, 5781, 5800, 5811, 5814, 5820, 5825, 5870, 5891, 5900, 7052, 7053, 7055, 7059, 7185, 7186, 7187, 7236, 7530.

### Methods

For each subject, the first four EPI images were discarded to allow for T1 equilibration. Outlier detection was based on a comparison of each volume with its two neighbors in a motion corrected time series. This was done by calculating the mean squared differences to the previous and the next volume. The smaller difference was used as deviation score for each volume. The scores were thresholded using the method of Hubert and van der Veeken (2008), which calculates a robust measure of skewness (medcouple, MC) and uses it for correcting the inter quartile range (IQR). For thresholding deviation scores, the IQR was multiplied by 1.5 and added to the 75th percentile (P75). Thresholds were derived for each session as well as globally for the whole data set. For detecting outlier sessions, statistics of each session's deviation scores (median, IQR, MC, and P75) were used as session characteristics and thresholded in both directions using the same method. The scores were included in the first level models of each subject as additional regressors.

Event-related BOLD responses were analyzed using the general linear model with a canonical hemodynamic response function as basic function and a high pass filter of 128 s. One separate event-related regressor with explicit onsets was modeled for each condition (*Individualized-OCD*, *Individualized-Neutral*, *Standardized-OCD*, *Standardized-Neutral*). Rating intervals were included as regressors of no interest. Both runs were modeled separately. Further, the six movement parameters obtained by the realignment procedure were introduced as covariates in the model. Contrasts of beta-estimates were calculated for each individual.

ROI masks were taken from the probabilistic Harvard-Oxford Cortical and Subcortical Structural Atlas (included in FSLView version 3.1; http://www.fmrib.ox.ac.uk/fsl/) if available. Masks for substantia nigra and dlPFC (BA48) are not available in this atlas and were taken from the WFUPICK atlas “brodmann areas+” (as provided with SPM8). Voxels were included in a mask if (1) the probability of belonging to the desired structure was higher than the probability for belonging to any other structure and (2) the probability for belonging to the desired structure was *p* > 0.25. Table A1 shows all used masks and their origins.

**Table A1:** **Overview over all ROI used in this study**.

**Structure**	**Name(s) in atlas**	**Atlas**
**AFFECTIVE LOOP**		
Orbitofrontal cortex	Frontal_orbital_cortex-maxprob-thr25	Harvard-Oxford
Ventral striatum	Accumbens-maxprob-thr25	Harvard-Oxford
Ventral pallidum	Pallidum-maxprob-thr25	Harvard-Oxford
Mediodorsal thalamus	Thalamus-maxprob-thr25	Harvard-Oxford
**SPATIAL/ATTENTIONAL LOOP**		
Angular gyrus	Angular_gyrus_maxprob-thr25	Harvard-Oxford
dlPFC	BA46	WFUPICK
N. Caudatus	Caudate-maxprob-thr25	Harvard-Oxford
Pallidum	Pallidum-maxprob-thr25	Harvard-Oxford
Thalamus	Thalamus-maxprob-thr25	Harvard-Oxford
Substantia nigra	Substantia nigra	WFUPICK

*The provided names apply bilaterally*.
